# Fire decline in dry tropical ecosystems enhances decadal land carbon sink

**DOI:** 10.1038/s41467-020-15852-2

**Published:** 2020-04-20

**Authors:** Yi Yin, A. Anthony Bloom, John Worden, Sassan Saatchi, Yan Yang, Mathew Williams, Junjie Liu, Zhe Jiang, Helen Worden, Kevin Bowman, Christian Frankenberg, David Schimel

**Affiliations:** 1 Division of Geological and Planetary Sciences, California Institute of Technology, Pasadena, CA 91125 USA; 2 Jet Propulsion Laboratory California Institute of Technology, Pasadena, CA 91101 USA; 3 School of Geosciences, University of Edinburgh, Edinburgh, EH9 3FF UK; 4 National Centre for Earth Observation, University of Edinburgh, Edinburgh, EH9 3FF UK; 5 School of Earth and Space Sciences, University of Science and Technology of China, 230026 Hefei, Anhui China; 6 National Center for Atmospheric Research, Boulder, CO USA

**Keywords:** Biogeochemistry, Climate-change ecology

## Abstract

The terrestrial carbon sink has significantly increased in the past decades, but the underlying mechanisms are still unclear. The current synthesis of process-based estimates of land and ocean sinks requires an additional sink of 0.6 PgC yr^−1^ in the last decade to explain the observed airborne fraction. A concurrent global fire decline was observed in association with tropical agriculture expansion and landscape fragmentation. Here we show that a decline of 0.2 ± 0.1 PgC yr^−1^ in fire emissions during 2008–2014 relative to 2001–2007 also induced an additional carbon sink enhancement of 0.4 ± 0.2 PgC yr^−1^ attributable to carbon cycle feedbacks, amounting to a combined sink increase comparable to the 0.6 PgC yr^−1^ budget imbalance. Our results suggest that the indirect effects of fire, in addition to the direct emissions, is an overlooked mechanism for explaining decadal-scale changes in the land carbon sink and highlight the importance of fire management in climate mitigation.

## Abstract (web summary)

In recent history the amount of carbon captured by terrestrial systems has increased, but the processes driving this process has remained poorly constrained. Here the authors use a global carbon model to show that a decrease in wildfires has caused the land carbon sink to increase in the past few decades.

## Introduction

Fire is an important disturbance agent in the terrestrial ecosystem, particularly in the dry tropics, tightly coupled with vegetation, climate, biogeochemical cycles, and human activities^1–3^. Climate plays a critical control on fire by regulating fuel load and conditions for fire ignition and spread^4–7^. However, human activities also affect fuel accumulation and fire risk and are responsible for most ignitions and all suppression efforts, thus they have a profound impact on the timing, frequency, extent, and intensity of fires^1,3,8^. With the rapid increase in human population and agricultural production in the last decades, many regions have transited from natural to human-dominated fire regimes^9^. A 25% decline in the global burned area (BA) from 1997 to 2015 has been observed combining multiple satellite data sets, with the most significant decreases in the savannas of Africa^10^. These declines are found to be correlated with agricultural expansion and landscape fragmentation^10,11^.

At the same time, a decrease in the fraction of anthropogenic CO_2_ emissions that remain in the atmosphere (airborne fraction, −2.2% per year) has been observed for the period 2002–2014^12^, despite continued increases in anthropogenic CO_2_ emissions^13^. However, processes explaining the terrestrial component of this increase—likely related to CO_2_ fertilization^12,14^, changing soil moisture and temperature regimes^15^, and land use and land cover change^16^—are still under debate. The Global Carbon Project (GCP) synthesizes observational and model-based flux estimates from multiple organizations and research groups around the world to report the Global Carbon Budget yearly^13^. Instead of treating the land sink as a residual term between the anthropogenic emissions and the atmospheric and ocean uptakes as historically being done, the most recent report provides explicit land sink estimates using an ensemble of land models that account for climate warming, CO_2_ fertilization, and land use change impacts, which result in a budget imbalance that requires an additional sink of ~0.6 PgC per year to explain the observed airborne fraction during the last decade^13^. Direct fire carbon emissions are often considered for global and regional carbon budgets^13,17,18^. In particular, deforestation fire and peatland burning are included in the land-use emission estimates^13,19^. However, impacts of wild fire decline on the subsequent ecosystem carbon cycling have not been well quantified, as prognostic fire models show large spreads in fire distribution and magnitude^10^ and fire modules in the land surface models included in the GCP synthesis are not explicitly guided by the observed BA changes^13^.

Under a dynamic equilibrium assumption, fire induces temporal changes in the carbon source and sink at yearly and decadal scale within one disturbance-recovery episode, but it has a negligible net effect on the long-term carbon budget because fire-induced carbon loss is eventually compensated by subsequent vegetation growth as the ecosystem recover toward equilibrium^20,21^. However, a shift in the fire regime could result in long-term carbon loss or gain if it leads to a different steady state of the carbon pools^20,21^. Here, we estimate changes in the land carbon sink attributable to the observed global BA decline over the last decade due to both direct (fuel combustion) and indirect (postfire ecosystem carbon cycle) impacts.

We use the CARbon DAta-MOdel fraMework (CARDAMOM^22^)—constrained by atmospheric and land-surface C observations throughout 2001–2014—to estimate the impacts of fire decline on the terrestrial carbon cycle. The schematic of CARDAMOM carbon pools and associated observational constraints are depicted in Supplementary Fig. 1. Specifically, fire occurrences are constrained by satellite-derived BA, and fire carbon emissions are derived as the product of BA, live biomass (leaf, labile, wood, and roots) and dead organic carbon stores (litter and soil organic carbon), as well as their associated combustion factors (percentages of fire loss relative to the total organic pool). In addition, biomass mortality rates are increased in the event of fire leading to a transfer of live biomass to dead organic carbon pools. The dynamics of the terrestrial carbon cycle are explicitly retrieved through a Bayesian model-data fusion estimation of key parameters for carbon cycle and initial states of carbon pools^22^. Assimilated datasets consist of fire carbon emissions inferred from atmospheric CO inversions assimilating MOPITT (Measurements Of Pollution In The Troposphere)^23^ and biome-specific CO to total carbon emission ratios^24^; satellite-derived observations of leaf area index (LAI)^25^, Gross Primary Production (GPP) variability inferred from solar-induced ﻿fluorescence (SIF)^26^, the spatial distribution of above-ground biomass^27^; inventory-derived global distribution of soil organic carbon (Harmonized World Soil Database, HWSD^28^). The combustion factors and fire-induced mortality rates at each model grid cell are optimized given the CO-derived total carbon emission estimates. We first estimate fire carbon emissions from 2001 to 2014 using the observation-constrained CARDAMOM analysis, and then quantify the impacts of the observed BA decline on the other carbon cycle processes using sensitivity simulations. Our results suggest a decline of 0.2 ± 0.1 PgC per year in fire emissions between the two period 2008–2014 and 2001–2007, which also induced an additional carbon sink enhancement of 0.4 ± 0.2 PgC per year attributable to carbon cycle feedbacks, amounting to a combined sink increase comparable to the 0.6 PgC per year budget imbalance.

## Results and discussion

### Observed declines in burned area and fire emissions

**Fig. 1:**
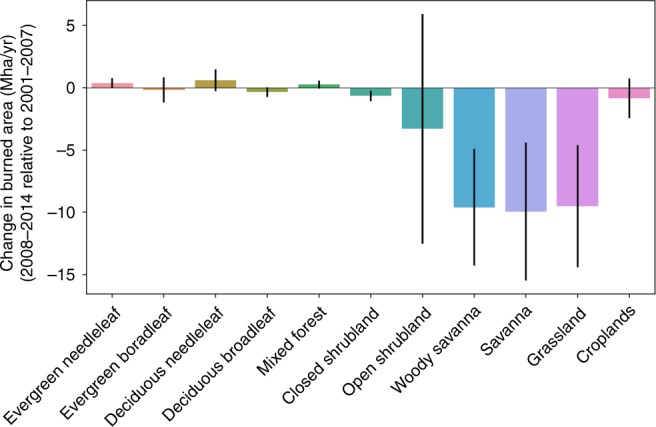
Burned Area Change between 2008–2014 and 2001–2007. Burned Area (BA) estimates are from GFED4 dataset (synthesis of MODIS MCD64A1) and land cover type from MODIS MCD12Q1 product. The error bars show the uncertainty of individual year-to-year differences between the two period 2008–2014 and 2001–2007.

Global BA decreased by 34 Mha per year (−9%) between 2001–2007 and 2008–2014 according to the Global Fire Emission Database (GFED4), and by 52 Mha per year (−10%) according to GFED4s that accounts for small fires using thermal active fire data in addition to the BA detected from changes in surface reflectance retrieved from the MODIS (Moderate Resolution Imaging Spectroradiometer) instrument^29,30^. BA declines occurred mostly in savanna (−10 Mha per year, −8%), woody savanna (−9.6 Mha per year, −7%), and grassland (−9.5 Mh per year, −21%) according to the MODIS land cover type^31^ (Fig. 1). The decrease of BA in the open shrubland is also large (−3.3 Mha per year, −15%) but has considerable interannual variations, whereas the decline in closed shrubland is significant but small in absolute magnitude (−0.7 Mha per year, −23%). In contrast, BA changes in forest—which have on average much lower fire frequencies—are relatively small in absolute magnitude and are associated with large interannual variations. The land cover types showing large BA declines have in general relatively short fire return times ranging from 1 year to a few years^3^, places showing significant decadal fire reductions are hence expected to experience changes in fire frequency and thus deviations from their typical fire disturbance-recovery trajectory. Additional BA dataset (ESA-CCI)^32^ that are also derived from MODIS instrument with a different algorithm shows a comparable spatial pattern as GFED4 and a decrease by 23 Mha per year (−6%) between the two periods (Supplementary Fig. 2). Relatively smaller declines are found when small fires are not explicitly considered. Here, we use GFED4s as the reference version for further analysis as it is important to account for variations in small fires to fully capture fire dynamics^29^.

**Fig. 2:**
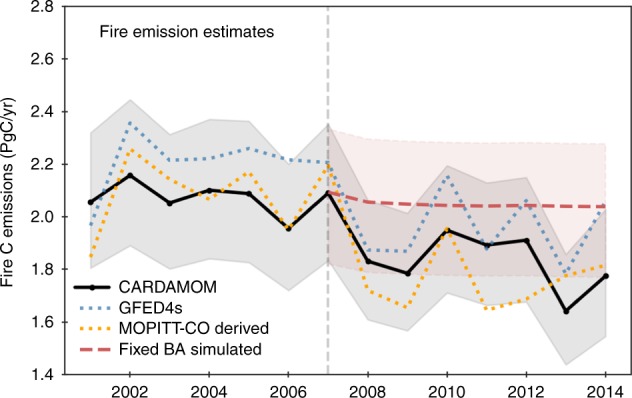
Global fire carbon emissions estimates for 2001–2014. The solid black line represents fire carbon emissions estimated by the ensemble median of CARDAMOM, and the shading describes the first and third quartiles using a Monte-Carlo sampling of the parameters. The dotted blue line shows GFED4s bottom-up estimates, and the dotted orange line shows the estimates derived from MOPITT CO inversion and biome-specific emission ratios between CO and total carbon. The dashed red line represents simulated fire emissions using the average burned area during 2001–2007.

Fire carbon emissions estimated using CARDAMOM amount to 2.1 ± 0.1 PgC per year (1PgC = 1e^15^ g Carbon) across the globe for the period 2001–2007 and 1.8 ± 0.1 PgC per year for the period 2008–2014 (Fig. 2, mean ± standard deviation across different years based on CARDAMOM median estimates). An average decrease rate of −1.5% per year is found over the period 2001–2014 (*p* = 0.01), with a faster decline of −1.8% per year (*p* = 0.02) during the latter half from 2007 to 2014 compared to the non-significant decline during the first half from 2001 to 2007 (−0.4%, *p* = 0.5). This decadal decrease is supported by the GFED bottom-up approach and the MOPITT CO-inversion top-down estimates (Supplementary Fig. 3). Reductions in fire emission between the two episodes are mostly contributed by North Africa (i.e., the Sahel and sub-Sahelian regions, −70 ± 9 TgC per year, −16%) (1 TgC = 1e^12^ g carbon), southern South America (−60 ± 20 TgC yr^−1^, −30%), northern South America (−53 ± 9 TgC per year, −36%), Southeast Asia (−55 ± 22 TgC per year, −24%), Australia (−23 ± 4 TgC per year, −16%), and Europe (−7.5 ± 0.8 TgC per year, −30%) (mean ± standard deviations among the three approaches).

### Impacts of fire decline on the subsequent carbon cycle

**Fig. 3:**
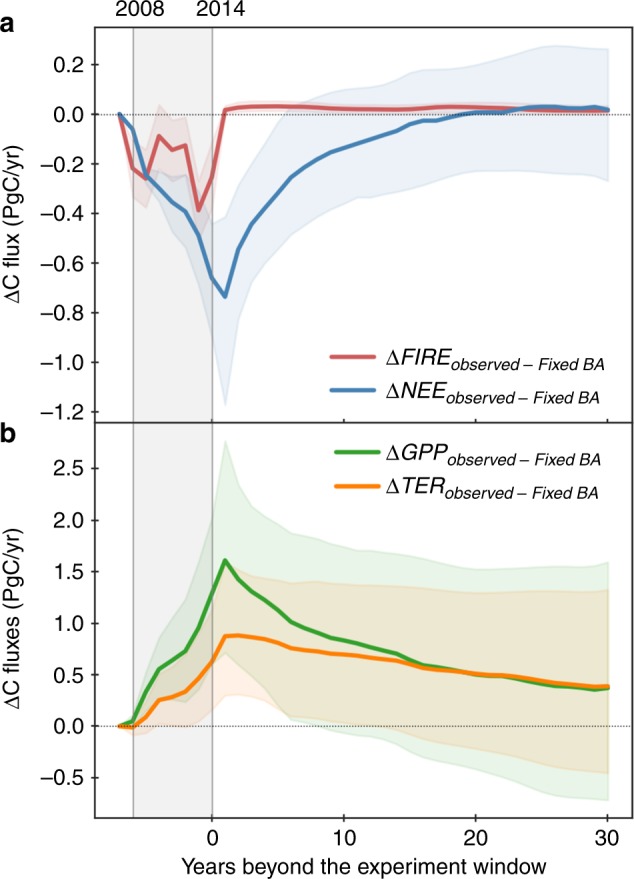
Impacts of different BA scenarios on the global carbon cycle. **a** Relative differences in the simulated global fire carbon emissions (ΔFIRE) and net ecosystem exchange (ΔNEE), and **b** Relative differences in the simulated global Gross Primary Productivity (ΔGPP) and Terrestrial Ecosystem Respiration (ΔTER) between the Observed BA and the hypothetical BA scenarios—Fixed BA. The lines show the model median and the shadings show the range between 1st and 3nd quartile. Different BA scenarios are only applied within the experiment window (2008–2014) as marked by the grey shade, resultant differences beyond 2014 represent legacy effects of different BA during 2008–2014. Actual meteorological data is used from 2001 to 2014, and the same forcing data is repeatedly used for all scenarios beyond 2014 including the Observed BA.

To quantify the impact of the observed BA decline between 2001–2007 and 2008–2014 on the carbon cycle, we performed a CARDAMOM control run with a constant 2008–2014 burned area, using average 2001–2007 burned area values. All else being equal, the simulated differences between the observation-retrieved carbon cycle states and fluxes (henceforth denoted as Observed BA) relative to the control run (henceforth denoted as Fixed BA) represent impacts of the Observed BA decline relative to the mean 2001–2007 fire levels. Fire emissions estimated using the Observed BA are 0.2 ± 0.1 PgC per year lower compared to the hypothetical case of Fixed BA averaged over 2008–2014 (Fig. 2, Fig. 3a). Beyond the 2008–2014 experiment window when all setups return to the same to evaluate the legacy effects of previous fire, differences in fire emissions between Observed BA and Fixed BA (ΔFIRE) reduced to +0.03 PgC per year averaged over 30 years. The slightly higher fire emissions associated with Observed BA result from larger fuel loads, because a lower 2008–2014 fire level had burned fewer organic matters and allowed more vegetation to regrow.

**Fig. 4:**
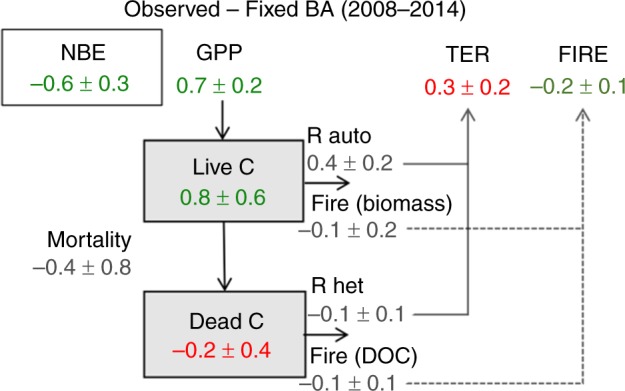
Differences in the major carbon fluxes and pools between the two scenarios. The 2008–2014 average differences between the Observed BA and Fixed BA scenarios are shown (Units: PgC per year). Increases in the land sink is denoted in green and decreases in the land sink is denoted in red. NEE = TER − GPP and NBE = NEE + FIRE; negative NBE values represent net fluxes from the atmosphere to the land. Uncertainties are estimated by MCMC simulations.

BA declines also result in a higher gross primary productivity relative to the control run (ΔGPP), as reduced fire occurrences allow more biomass to grow and hence an enhanced photosynthetic capacity (Fig. 3b). The increase in ΔGPP due to BA decline results in an increase in the live biomass (Fig. 4), and an increase in turnover-induced dead carbon pool inputs; conversely, the BA decline results in a reduction in fire-induced mortality and hence a decline in dead carbon pool inputs. Due to the competing mortality processes the net dead organic carbon stock changes are more uncertain (Fig. 4). However, the overall increase in the terrestrial ecosystem respiration (ΔTER) is a factor of two smaller than ΔGPP (Fig. 3b), thus resulting in a significant net ecosystem exchange (ΔNEE) reduction relative to the case of Fixed BA averaged over 2008–2014 (ΔNEE = ΔTER−ΔGPP, with positive values representing net fluxes from the land to the atmosphere). The indirect effect of fire decline on NEE is roughly twice the magnitude of the corresponding direct impacts on fire emissions.

Unlike ΔFIRE that show immediate responses to concurrent BA changes, ΔNEE increases gradually within the 2008–2014 experiment window as the cumulative differences in BA (ΔBA) grow (Fig. 3a). The magnitude of ΔNEE fluxes starts to decrease with a 1-year lag beyond the experiment window (i.e. response to ΔBA of previous years), showing a near exponential decline of the legacy effects (Fig. 3a). The average ΔNEE during the first 5 years after BA perturbation is comparable to average effect within the experiment window (−0.4 ± 0.1 PgC per year), but they reduce to <50% and 10%, respectively, for the 6-to-10-year and 11-to-20-year windows (Fig. 3a). When accounting for fires, a neutral Net Biome Exchange (NBE = TER + FIRE-GPP) is reached within 18 years for the global average, more rapidly in South America and Africa (Supplementary Fig. 4), where a dominant contribution came from savanna ecosystem that are associated with a relatively short carbon residence time compared to forest^21,22,33^.

**Fig. 5:**
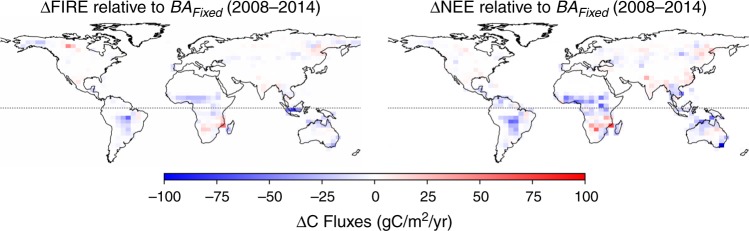
Distribution of differences in fire and NEE fluxes between scenarios. Differences in the CARDAMOM simulated fire and NEE fluxes between the Observed BA and the Fixed BA scenarios averaged between 2008 and 2014.

The GPP enhancement (and associated NEE reduction) is mostly attributable to the same regions exhibiting significant fire emission reductions (Fig. 5), namely the Sahel region in North Africa and dry sub-tropics in South America. In these dry tropical areas, positive trends are observed in the dry season enhanced vegetation index (EVI) and near-infrared reflectance of vegetation (NIRv) over the study period 2001–2014 (Supplementary Fig. 5), which is in agreement with our simulated responses in GPP to the burned area reduction. Besides, forest expansion and woody encroachment in the savannahs in Central and Western Africa are observed from space^34^, consistent with our simulated responses to BA decline (Fig. 5). The regions in the dry tropics showing significant responses in simulated NEE are also generally in line with areas where satellite-based aboveground biomass estimates indicate net gains^35^. We note that the estimated increase in GPP due to reduced fire did not account for nutrient limitation^36^ or grazing^37^, thus our estimation might be on the higher bound and future studies are needed to better understand the long-term effect to account for those factors along with vegetation successions^38^. Nevertheless, our simulated gains in the above-ground biomass after 7 years of fire reduction are in line with site-level observations that show substantial accumulation of woody biomass relative to 1-year fire frequency sites (+220 gC m^−2^) or relative to 3-year fire frequency sites (+130 gC m^−2^) after long-term fire exclusion in a tropical savanna ecosystem^39^, as well as a +160 gC m^−2^ increase in grass biomass compared to the 1-yr fire frequency site (Supplementary Fig. 6). This site-level comparison shows the estimated carbon enhancement are realistic at a process level. The simulated responses are also comparable with boreal fire studies showing a decrease of net primary production (NPP) by 60–260 gC m^−2^ per year after fire disturbance^40^.

### Implications for the global carbon cycle

**Fig. 6:**
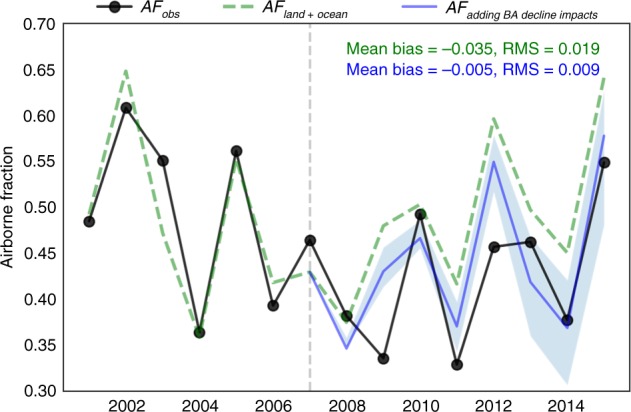
Estimated impacts of fire decline on airborne fraction. The impacts of fire decline between 2008–2014 and 2001–2007 are evaluated here. Emissions from Fossil fuel (*E*_FF_) and from land use change (E_LUC_), atmospheric CO_2_ growth rate (*G*_ATM_), and process-based sink estimates for ocean (S_OCEAN_) and land (S_LAND_) are derived from GCP 2018 estimates to determine the airborne fraction (each component shown in Supplementary Fig. 10. AF_obs_ = *G*_ATM_/(*E*_FF_ + E_LUC_), showing the observed *G*_ATM_ relative to the total emissions from *E*_FF_ and *E*_LUC_; AF_land+ocean_ = 1 − (*S*_OCEAN_ + *S*_LAND_)/(*E*_FF_ + *E*_LUC_), representing the portion of AF variations that can be explained by current process-based estimates of land and ocean sinks; AF_+BA Decline_ = 1 − (*S*_OCEAN_ + *S*_LAND_ − ΔFIRE − ΔNEE)/(*E*_FF_ + *E*_LUC_), representing the adjusted AF by adding for our estimates of BA decline impacts relative to the mean 2001–2007 BA. Note that the adaptations to 2015 are only due to the legacy effect of the period 2008–2014.

Accounting for our estimation of the fire decline impacts on the global carbon cycle relative to the case of Fixed BA at average 2001–2007 fire level, we could better explain the observed airborne fraction building on estimates of the other components from the GCP (see Methods). The adjusted airborne fraction estimates accounting for both direct and indirect effects of fire decline relative to the 2002–2007 mean significantly reduces the mean bias of the initial GCP estimates by 86% and the RMS by 53% reduction (Fig. 6). This improvement suggests that the enhancement of the land carbon sink due to fire reduction might have contributed to the global carbon budget with a magnitude comparable to the current estimates of the imbalance. It could have played a significant role in the recent terrestrial carbon sink increase, in addition to the widely recognized impacts of climate warming, CO_2_ fertilization, and land use change as addressed in GCP^13^, as well as other processes that are not explicitly accounted for. While land use change emissions do account for fire emissions, in particular, those related to deforestation and cropland conversion, and typical subsequent recovery^13,16,19^, the mechanisms we highlight here include the explicit representation of feedbacks between reduced fires and increased leaf area, potential limitations induced by additional growth potential through water availability, and subsequent impacts on heterotrophic respiration. Further investigation into the relative impacts of these processes is critical to improve understanding and reduce uncertainty on the response of ecosystems to reduced fire activity. We also note that although CARDAMOM results explicitly represent the role of parametric uncertainty on the reported increases in GPP and NEE, it is necessary for future efforts to investigate the role of model structural uncertainties.

Here, we focus on the impacts of fire decline between the two periods, 2001–2007 and 2008–2014, hence the legacy effects of past fire dynamics (those before 2007) are not explicitly addressed—even though their impacts are implicitly included in the carbon cycle parameters inferred from satellite data using CARDAMOM. Their impacts would have been identical for our sensitivity simulations, thus not impacting the results deduced from differences between the BA scenarios. Trace gas mixing ratios in ice cores and charcoal data indicates a much higher biomass burning emissions over the past millennium compared to the contemporary level^41,42^. African fires, contributing to around 60% of the global fire carbon emissions, have declined since the 1950s^9^. A recent model study showed that increasing population densities and cropland area have decreased global BA since the 1930s and result in a significant reduction in global fire emissions (0.13 PgC per year for the period 1960–2009)﻿^43^, but the indirect impacts on NEE in addition to fire emissions are not analyzed explicitly. The much larger magnitude of the indirect, lagged NBE effect, as we show here, due to fire decline relative to its direct impacts on fire emission implies an even larger contribution of fire declines to the global land sink increase during the past decades. Missing this important mechanism in explaining the global land carbon sink increase over the past decades could lead to an overestimation of other processes, such as CO_2_ fertilization^44,45^, and thus significant biases in future carbon-climate feedback projections. The indirect effect of the global burned area decline is an overlooked mechanism that may explain some of the land carbon sink increase in recent decades. This mechanism is likely effective at a decadal time scale, if a lower fire level is sustained, till the ecosystem reaches a steady state but unlikely to continue indefinitely as climate projections suggest increased fire risk^8^ and extreme droughts associated with El Niño events could result in non-linear fire responses^5,18^. Therefore, fire management is an important strategy for terrestrial carbon storage and thus climate mitigation.

## Methods

### Satellite-derived burned area and bottom-up fire emissions

We use satellite-derived burned area (BA) from the Global Fire Emissions Database (GFED)^30,46^ and the ESA-CCI product^32^ at a spatial resolution of 0.25° and a temporal resolution of monthly. We include two versions from GFED: GFED4 based on changes in the surface reflectance, and GFED4s that, in addition to the GFED4 BA, account for small fires using active fire information to extend the detection limit^47^. We focus on the period from 2001 to 2014, during which the BA are consistently retrieved from MODIS and the atmospheric CO retrievals are available from MOPITT. GFED4s BA is used as the reference version in this study, while the rests are used for sensitivity tests. GFED also provides gridded monthly fire emissions from multiple fire tracers using a bottom-up approach^2,46^. CO emission estimates from GFED3 are used as the prior for fire emissions in our atmospheric inversion described below.

### Atmospheric top-down fire emissions estimates

Complementary to the ground fire features derived from satellite, trace gases emitted from biomass burning could provide valuable top-down constraints to fire emission estimates, in particular CO, because it has a relatively simple source structure (mainly from fossil fuel and biomass burning with relatively small spatial colocations) and a lifetime of a few weeks allowing the track of the transport from its source regions^48^. Using a two-step inversion system that combines a sequential Kalman filter to optimize boundary conditions and a variational assimilation system to optimize fluxes, we assimilate MOPITT CO retrievals (version 6^49^) with GeosChem to optimize monthly CO emissions from fire and fossil fuel, and additional sources from hydrocarbon oxidation (details are documented in Jiang et al.^23^). The version of GeosChem we use here has a spatial resolution of 4° × 5° and a vertical resolution of 47 levels^23^. We convert MOPITT-derived fire CO emission into fire carbon emission using biome-specific ratios of emission factors between CO and the total carbon following GFED4. Uncertainties in the CO inversion and the ratio between emission factors are propagated into the uncertainty of CO-derived fire carbon emission estimates following the method in Worden et al.^50^. The derived fire carbon emissions and associated uncertainties are used to constraint CARDAMOM fire emissions described below.

### CARDAMOM: data constrained carbon cycle model

CARDAMOM represents six carbon pools (foliar, labile, wood, fine roots, litter, and soil carbon) and one plant-available water pool in each model grid, and simulates the processes controlling their dynamic evolutions in time^22,51^ (Supplementary Fig. 1). The forcing data consist of monthly meteorology reanalysis (ERA- interim) from European Centre for Medium-Range Weather Forecasts (ECMWF) and BA from GFED4s. The total carbon input is represented by the gross primary production (GPP), which is a function of meteorology and the photosynthetic capacity depending on foliar carbon pool; autotrophic respiration (Ra) is a function of GPP and temperature; the net primary production (NPP) is then allocated into the four live biomass pools. Plant mortality is expressed by the turnover time of each carbon pool and organic matters moved into litter or soil carbon pools are subject to further decomposition as a function of temperature and moisture (Rh). Fire is introduced by prescribed BA, causing the combustion of live biomass and dead organic matters and an increase in mortality rate. Key model parameters controlling the carbon cycle (photosynthesis, phenology, allocation, and turnover rates) and fire-related processes (combustion factors for foliar, structural, litter and soil C pools, as well as a fire resilience factor) are optimized within each grid at a 4° × 5° resolution, the same resolution as the MOPITT-CO inversion, using a Metropolis-Hastings Markov Chain Monte Carlo Approach^22^. The parameters are not distinguished by plant functional types (please see details in Bloom et al.^22^; Bloom et al.^51^).

### Deducing fire decline impacts on the airborne fraction

We convert our estimated ΔFIRE and ΔNEE fluxes between the Observed BA and the Fixed BA scenarios into equivalent changes in the airborne fraction (AF). The other budget terms are adopted from the most recent GCP global carbon budget)^13^. For each year, GCP synthesize CO_2_ emissions from fossil fuel and industry using energy statistics (*E*_FF_), land-use change emissions based on bookkeeping models (*E*_LUC_), and ocean (*S*_OCEAN_) and terrestrial carbon sinks (*S*_LAND_) based on the state-of-the-art process models (Supplementary Fig. 7). The atmospheric growth rate is directly determined by the atmospheric CO_2_ observations (*G*_ATM_). Ideally, if every component is accurately estimated: *E*_FF_ + *E*_LUC_ = *G*_ATM_ + *S*_OCEAN_ + *S*_LAND_, the observed airborne fraction, AF_obs_ = *G*_ATM_/(*E*_FF_ + *E*_LUC_), would match perfectly the process-explained variations, AF_land+ocean_ = 1 − (*S*_OCEAN_ + *S*_LAND_)/(*E*_FF_ + *E*_LUC_). However, due to imperfect understanding of the contemporary carbon cycle and model representation, there is a mismatch. We thus include our estimated fire impacts on ΔFIRE and ΔNEE to evaluate the updated attribution, AF_+BA Decline_ = 1 − (*S*_OCEAN_ + *S*_LAND_ − ΔFIRE − ΔNEE)/(*E*_FF_ + *E*_LUC_).

## Supplementary information

Supplementary Information

Peer Review File

## Data Availability

All data and CARDAMOM code are available upon request from the corresponding authors.
