# Supplementary material for: Fire decline in dry tropical ecosystems enhances decadal land carbon sink
Source: Nat Commun. 2020 Apr 20;11:1900. doi: 10.1038/s41467-020-15852-2 (PMC7170937; doi:10.1038/s41467-020-15852-2)
Supplement: Supplementary file 1 — Supplementary Information [file 41467_2020_15852_MOESM1_ESM.pdf]

*Supplementary Information for "***Fire decline in the dry tropical ecosystems enhances decadal land carbon sink" by Yin et al.**

Supplementary Figures

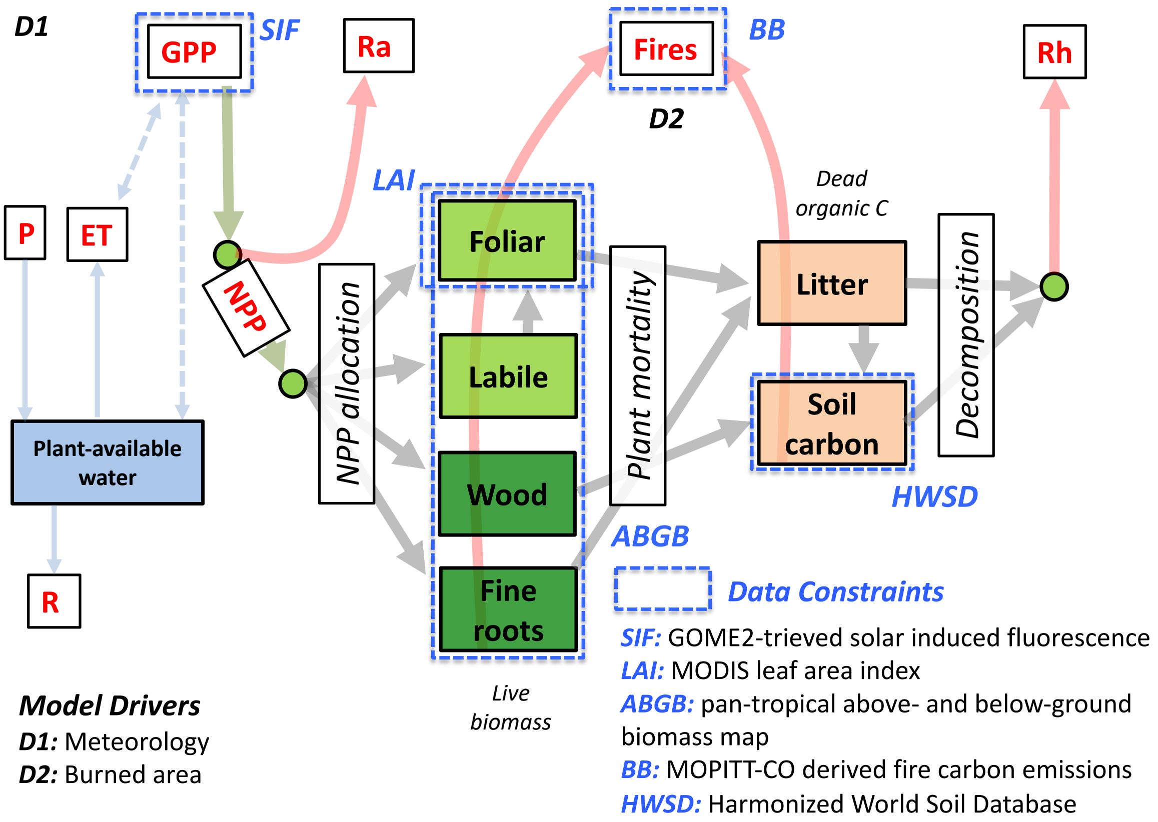

Supplementary Figure 1. Schematic of CARDAMOM (CARbon DAta-MOdel framework). The forcing data are noted as D1 and D2. Datasets assimilated in this study are noted in blue boxes with dashed lines.

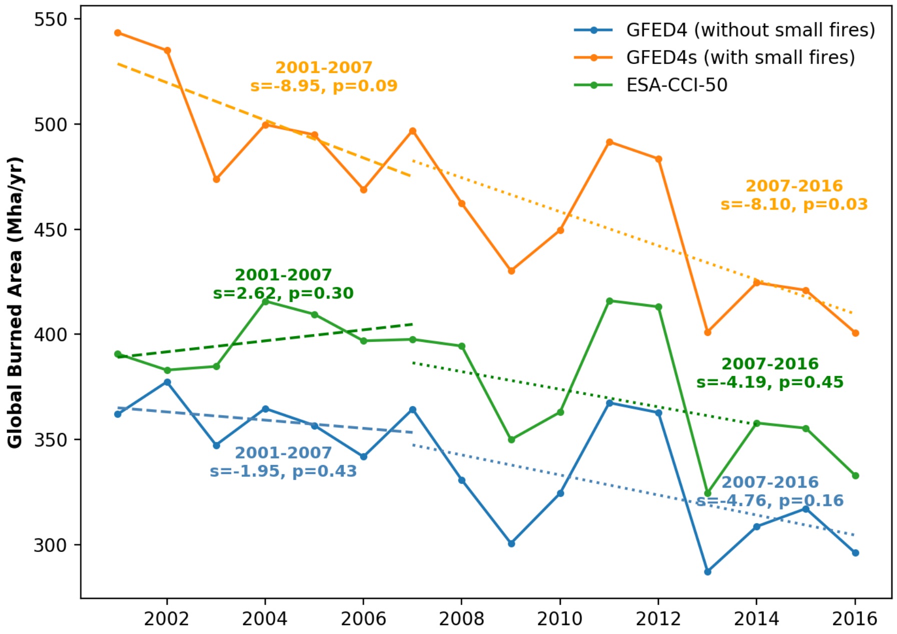

Supplementary Figure 2. Global Burned area. Different estimation from GFED4 without small fires, GFED4s including small fires, and ESA-CCI burned area dataset.

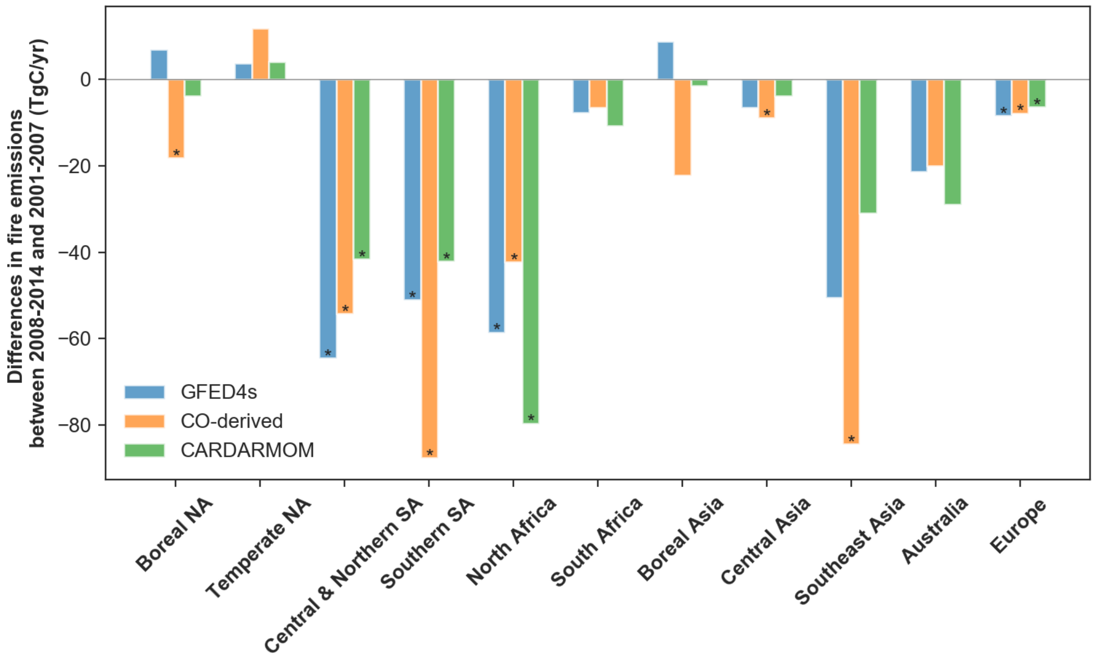

Supplementary Figure 3. Differences in regional fire carbon emissions between 2008-2014 and 2001-2007. NA represents North America and SA represents South America. The asterisks denote that differences across these two periods are statistically significant at a 90% confidence level.

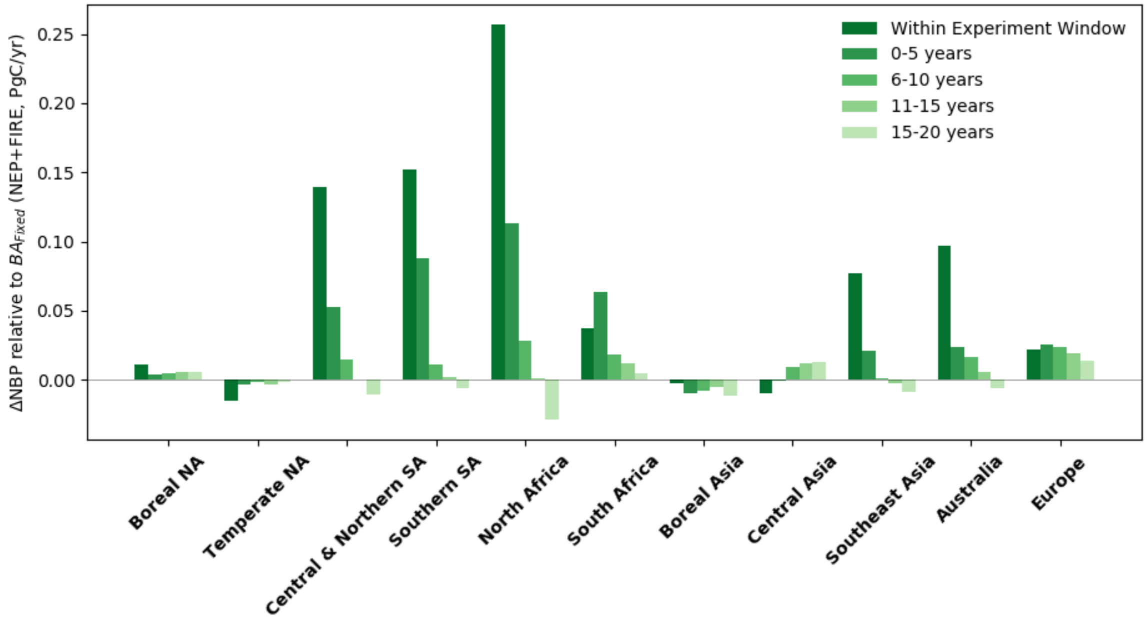

Supplementary Figure 4. Relative differences in NBP (NEP - FIRE) simulated with Observed relative to Fixed BA. In each sub-region annual average of five different stages are shown: within the experiment window 2008-2014 and legacy effects for the first 5 years, 6-10 years, 11-15 years, and 15-20 years.

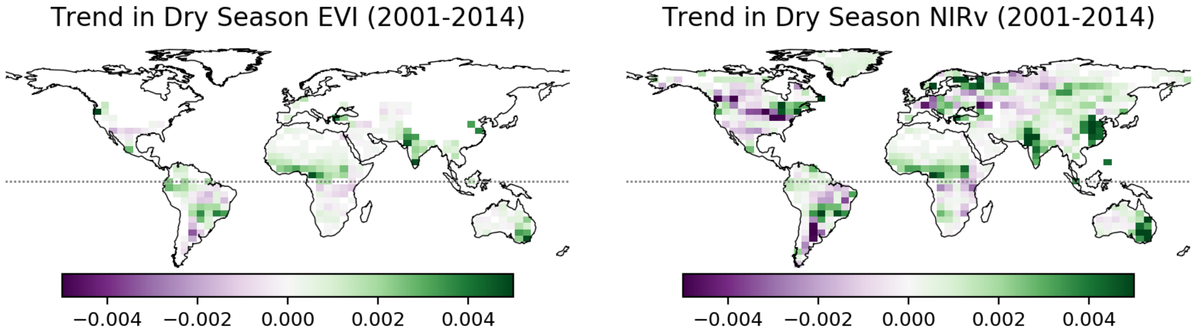

Supplementary Figure 5. Trends in dry season vegetation indexes derived from MODIS. Dry season defined by the month of the lowest precipitation for the 2001-2014 period.

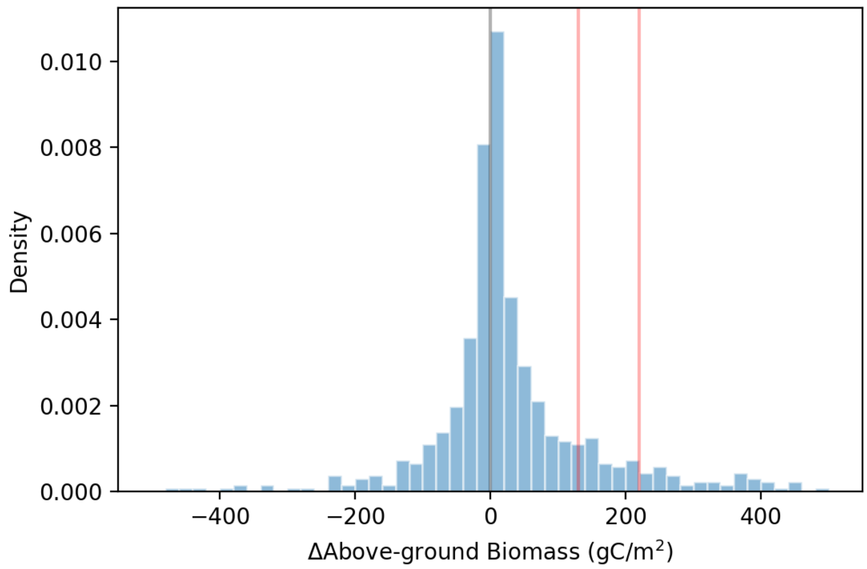

Supplementary Figure 6. Histogram of simulated differences in the above-ground biomass between Observed and Fixed BA. The simulated state in 2014 at the end of the experiment window are shown. Positive values suggest larger biomass accumulation relative to the case if fixed BA at the early 2000s level. The two red vertical lines mark site level observation of woody biomass accumulation in the case of long-term fire exclusion relative to 1-yr (+220 gC m^{-2}) and 3-yr (+130 gC m^{-2}) fire frequency plots in a tropical savanna ecosystem as documented in Pellegrini et al., (2015) [1].

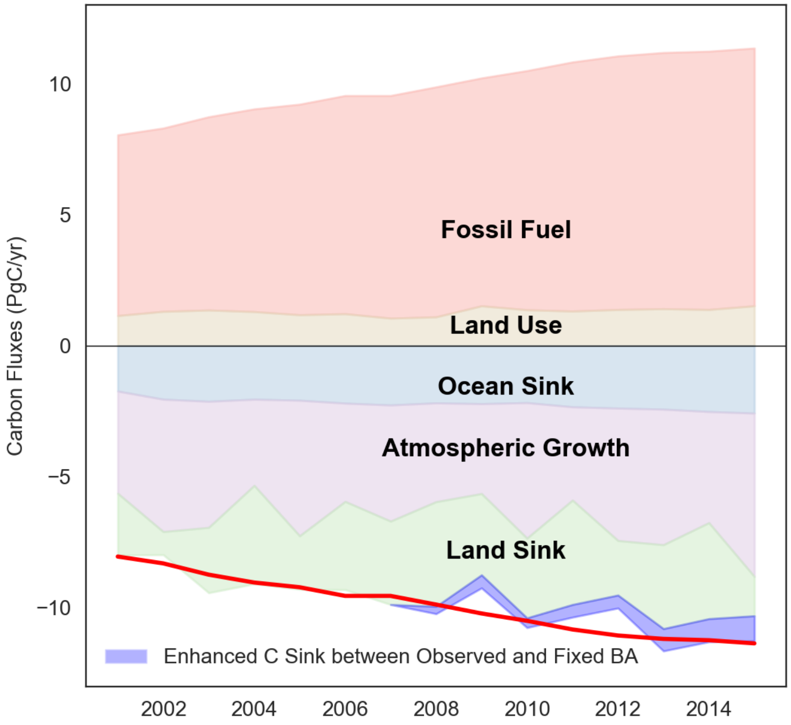

Supplementary Figure 7. Combined components of the 2002-2015 global carbon budget from the recent GCP report (Le Quéré et al., 2018 [2]). Each color represents a sector as noted. The red line shows the reflection of the total emissions, and differences between the red line and the added sink of the ocean, atmosphere, and land represent the budget imbalance. CARDAMOM estimated land sink increase due to fire decline relative to the 2001-2007 average are shown in blue.

References

1. Pellegrini, A. F. A., Hedin, L. O., Staver, A. C. & Govender, N. Fire alters ecosystem carbon and nutrients but not plant nutrient stoichiometry or composition in tropical savanna. *Ecology* **96**, 1275–1285 (2015).
2. Le Quéré, C. *et al.* Global Carbon Budget 2017. *Earth Syst. Sci. Data* **10**, 405–448 (2018).
