# Supplementary material for: Fire decline in dry tropical ecosystems enhances decadal land carbon sink
Source: Nat Commun. 2020 Apr 20;11:1900. doi: 10.1038/s41467-020-15852-2 (PMC7170937; doi:10.1038/s41467-020-15852-2)
Supplement: Supplementary file 2 — Peer Review File [file 41467_2020_15852_MOESM2_ESM.pdf]

Editorial Note: This manuscript has been previously reviewed at another journal that is not operating a transparent peer review scheme. This document only contains reviewer comments and rebuttal letters for versions considered at *Nature Communications* .

REVIEWERS' COMMENTS:

Reviewer #1 (Remarks to the Author):

The authors have addressed my concerns in this version. I am in favor of publishing the manuscript.

Reviewer #3 (Remarks to the Author):

Compared to the previous version, the authors have made significant efforts to improve their study. In this revision, they demonstrated the validity of their vegetation model. They also provided strong evidences to show how the reductions in fire activity contribute to the increased GPP or vegetation index. I would be happy to see the publication of this study, which brings new insights to the carbon cycle community.
